# Supplementary material for: Hybrid Models and Biological Model Reduction with PyDSTool
Source: PLoS Comput Biol. 2012 Aug 9;8(8):e1002628. doi: 10.1371/journal.pcbi.1002628 (PMC3415397; doi:10.1371/journal.pcbi.1002628)
Supplement: Text S4 — Complete source code for the PyDSTool package (version 0.88.120504). Includes API documentation and help files linking to web pages. This file is identical to the current public release on Sourceforge.net. (ZIP) [file pcbi.1002628.s004.zip › PyDSTool/html/PyDSTool.PyCont.BifPoint-pysrc.html]

xml version="1.0" encoding="ascii"?


PyDSTool.PyCont.BifPoint


| Home | Trees | Indices | Help | PyDSTool |
| --- | --- | --- | --- | --- |

Package PyDSTool ::
Package PyCont ::
Module BifPoint

[hide private]

[frames] | no frames]

# Source Code for Module PyDSTool.PyCont.BifPoint

```
  1  """ Bifurcation point classes.  Each class locates and processes bifurcation points.
 
  2  
 
  3      Drew LaMar, March 2006
 
  4  """ 
  5  
 
  6  from misc import * 
  7  from PyDSTool.common import args 
  8  from TestFunc import DiscreteMap, FixedPointMap 
  9  
 
 10  from numpy import Inf, NaN, isfinite, r_, c_, sign, mod, mat, \
 
 11      subtract, divide, transpose, eye, real, imag, \
 
 12      conjugate, average 
 13  from scipy import optimize, linalg 
 14  from numpy import dot as matrixmultiply 
 15  from numpy import array, float, complex, int, float64, complex64, int32, \
 
 16      zeros, divide, subtract, reshape, argsort, nonzero 
 17  
 
 18  #####
 
 19  _classes = ['BifPoint', 'BPoint', 'BranchPoint', 'FoldPoint', 'HopfPoint',
 
 20              'BTPoint', 'ZHPoint', 'CPPoint', 'DHPoint', 'GHPoint', 'LPCPoint',
 
 21              'PDPoint', 'NSPoint', 'SPoint'] 
 22  
 
 23  __all__ = _classes 
 24  #####
 
 25  
 


26 -class BifPoint(object):


27 -    def __init__(self, testfuncs, flagfuncs, label='Bifurcation', stop=False):


28          self.testfuncs = [] 
 29          self.flagfuncs = [] 
 30          self.found = [] 
 31          self.label = label 
 32          self.stop = stop 
 33          self.data = args() 
 34  
 
 35          if not isinstance(testfuncs, list): 
 36              testfuncs = [testfuncs] 
 37          if not isinstance(flagfuncs, list): 
 38              flagfuncs = [flagfuncs] 
 39  
 
 40          self.testfuncs.extend(testfuncs) 
 41          self.flagfuncs.extend(flagfuncs) 
 42          self.tflen = len(self.testfuncs)

 43  
 


44 -    def locate(self, P1, P2, C):


45          pointlist = [] 
 46  
 
 47          for i, testfunc in enumerate(self.testfuncs): 
 48              if self.flagfuncs[i] == iszero: 
 49                  for ind in range(testfunc.m): 
 50                      X, V = testfunc.findzero(P1, P2, ind) 
 51                      pointlist.append((X,V)) 
 52  
 
 53          X = average([point[0] for point in pointlist], axis=0) 
 54          V = average([point[1] for point in pointlist], axis=0) 
 55          C.Corrector(X,V) 
 56  
 
 57          return X, V

 58  
 


59 -    def process(self, X, V, C):


60          data = args() 
 61          data.X = todict(C, X) 
 62          data.V = todict(C, V) 
 63  
 
 64          self.found.append(data)

 65  
 


66 -    def info(self, C, ind=None, strlist=None):


67          if ind is None: 
 68              ind = range(len(self.found)) 
 69          elif isinstance(ind, int): 
 70              ind = [ind] 
 71  
 
 72          if C.verbosity >= 1: 
 73              print self.label + ' Point found ' 
 74          if C.verbosity >= 2: 
 75              print '========================== ' 
 76              for n, i in enumerate(ind): 
 77                  print n, ': ' 
 78                  Xd = self.found[i].X 
 79                  for k, j in Xd.iteritems(): 
 80                      print k, ' = ', j 
 81                  print '' 
 82                  if hasattr(self.found[i], 'eigs'): 
 83                      print 'Eigenvalues = \n' 
 84                      for x in self.found[i].eigs: 
 85                          print '     (%f,%f)' % (x.real, x.imag) 
 86                      print '\n' 
 87                  if strlist is not None: 
 88                      for string in strlist: 
 89                          print string 
 90                      print ''

 91  
 
 92  
 


93 -class SPoint(BifPoint):


94      """Special point that represents user-selected free parameter values.""" 


95 -    def __init__(self, testfuncs, flagfuncs, stop=False):


96          BifPoint.__init__(self, testfuncs, flagfuncs, 'S', stop=stop)

 97  
 


98 -    def process(self, X, V, C):


99          BifPoint.process(self, X, V, C) 
100          self.info(C, -1) 
101          return True

102  
 
103  
 


104 -class BPoint(BifPoint):


105      """Special point that represents boundary of computational domain.""" 


106 -    def __init__(self, testfuncs, flagfuncs, stop=False):


107          BifPoint.__init__(self, testfuncs, flagfuncs, 'B', stop=stop)

108  
 


109 -    def locate(self, P1, P2, C):


110          # Find location that triggered testfunc and initialize testfunc to that index
 
111          val1 = (P1[0]-self.testfuncs[0].lower)*(self.testfuncs[0].upper-P1[0]) 
112          val2 = (P2[0]-self.testfuncs[0].lower)*(self.testfuncs[0].upper-P2[0]) 
113          ind = nonzero(val1*val2 < 0) 
114          self.testfuncs[0].ind = ind 
115          self.testfuncs[0].func = self.testfuncs[0].one 
116  
 
117          X, V = BifPoint.locate(self, P1, P2, C) 
118  
 
119          # Set testfunc back to monitoring all
 
120          self.testfuncs[0].ind = None 
121          self.testfuncs[0].func = self.testfuncs[0].all 
122  
 
123          return X, V

124  
 


125 -    def process(self, X, V, C):


126          BifPoint.process(self, X, V, C) 
127  
 
128          self.info(C, -1) 
129  
 
130          return True

131  
 


132 -    def info(self, C, ind=None):


133          if ind is None: 
134              ind = range(len(self.found)) 
135          elif isinstance(ind, int): 
136              ind = [ind] 
137  
 
138          BifPoint.info(self, C, ind)

139  
 
140  
 
141  
 


142 -class BranchPoint(BifPoint):


143      """Currently only works for EquilibriumCurve""" 


144 -    def __init__(self, testfuncs, flagfuncs, stop=False):


145          BifPoint.__init__(self, testfuncs, flagfuncs, 'BP', stop=stop)

146  
 


147 -    def __locate_newton(self, X, C):


148          """x[0:self.dim] = (x,alpha)
 
149             x[self.dim] = beta
 
150             x[self.dim+1:2*self.dim] = p
 
151          """ 
152          J_coords = C.CorrFunc.jac(X[0:C.dim], C.coords) 
153          J_params = C.CorrFunc.jac(X[0:C.dim], C.params) 
154  
 
155          return r_[C.CorrFunc(X[0:C.dim]) + X[C.dim]*X[C.dim+1:], \
 
156                    matrixmultiply(transpose(J_coords),X[C.dim+1:]), \
 
157                    matrixmultiply(transpose(X[C.dim+1:]),J_params), \
 
158                    matrixmultiply(transpose(X[C.dim+1:]),X[C.dim+1:]) - 1]

159  
 


160 -    def locate(self, P1, P2, C):


161          # Initiliaze p vector to eigenvector with smallest eigenvalue
 
162          X, V = P1 
163  
 
164          J_coords = C.CorrFunc.jac(X, C.coords) 
165  
 
166          W, VL = linalg.eig(J_coords, left=1, right=0) 
167          ind = argsort([abs(eig) for eig in W])[0] 
168          p = real(VL[:,ind]) 
169  
 
170          initpoint = zeros(2*C.dim, float) 
171          initpoint[0:C.dim] = X 
172          initpoint[C.dim+1:] = p 
173  
 
174          X = optimize.fsolve(self.__locate_newton, initpoint, C) 
175          self.data.psi = X[C.dim+1:] 
176          X = X[0:C.dim] 
177  
 
178          return X, V

179  
 


180 -    def process(self, X, V, C):


181          BifPoint.process(self, X, V, C) 
182  
 
183          # Finds the new branch
 
184          J_coords = C.CorrFunc.jac(X, C.coords) 
185          J_params = C.CorrFunc.jac(X, C.params) 
186          A = r_[c_[J_coords, J_params], [V]] 
187          W, VR = linalg.eig(A) 
188          W0 = [ind for ind, eig in enumerate(W) if abs(eig) < 5e-5] 
189          V1 = real(VR[:,W0[0]]) 
190  
 
191          H = C.CorrFunc.hess(X, C.coords+C.params, C.coords+C.params) 
192          c11 = matrixmultiply(self.data.psi,[bilinearform(H[i,:,:], V, V) for i in range(H.shape[0])]) 
193          c12 = matrixmultiply(self.data.psi,[bilinearform(H[i,:,:], V, V1) for i in range(H.shape[0])]) 
194          c22 = matrixmultiply(self.data.psi,[bilinearform(H[i,:,:], V1, V1) for i in range(H.shape[0])]) 
195  
 
196          beta = 1 
197          alpha = -1*c22/(2*c12) 
198          V1 = alpha*V + beta*V1 
199          V1 /= linalg.norm(V1) 
200  
 
201          self.found[-1].eigs = W 
202          self.found[-1].branch = todict(C, V1) 
203  
 
204          self.info(C, -1) 
205  
 
206          return True

207  
 


208 -    def info(self, C, ind=None):


209          if ind is None: 
210              ind = range(len(self.found)) 
211          elif isinstance(ind, int): 
212              ind = [ind] 
213  
 
214          strlist = [] 
215          for n, i in enumerate(ind): 
216              strlist.append('branch angle = ' + repr(matrixmultiply(tocoords(C, self.found[i].V), \
 
217                  tocoords(C, self.found[i].branch)))) 
218  
 
219          BifPoint.info(self, C, ind, strlist)

220  
 
221  
 
222  
 


223 -class FoldPoint(BifPoint):


224 -    def __init__(self, testfuncs, flagfuncs, stop=False):


225          BifPoint.__init__(self, testfuncs, flagfuncs, 'LP', stop=stop)

226  
 


227 -    def process(self, X, V, C):


228          BifPoint.process(self, X, V, C) 
229  
 
230          # Compute normal form coefficient
 
231          # NOTE: These are for free when using bordering technique!)
 
232          # NOTE: Does not agree with MATCONT output! (if |p| = |q| = 1, then it does)
 
233          J_coords = C.CorrFunc.jac(X, C.coords) 
234          W, VL, VR = linalg.eig(J_coords, left=1, right=1) 
235          minW = min(abs(W)) 
236          ind = [(abs(eig) < minW+1e-8) and (abs(eig) > minW-1e-8) for eig in W].index(True) 
237          p, q = real(VL[:,ind]), real(VR[:,ind]) 
238          p /= matrixmultiply(p,q) 
239  
 
240          B = C.CorrFunc.hess(X, C.coords, C.coords) 
241          self.found[-1].a = abs(0.5*matrixmultiply(p,[bilinearform(B[i,:,:], q, q) for i in range(B.shape[0])])) 
242          self.found[-1].eigs = W 
243  
 
244          numzero = len([eig for eig in W if abs(eig) < 1e-4]) 
245          if numzero > 1: 
246              if C.verbosity >= 2: 
247                  print 'Fold-Fold!\n' 
248              del self.found[-1] 
249              return False 
250          elif numzero == 0: 
251              if C.verbosity >= 2: 
252                  print 'False positive!\n' 
253              del self.found[-1] 
254              return False 
255  
 
256          if C.verbosity >= 2: 
257              print '\nChecking...' 
258              print '  |q| = %f' % linalg.norm(q) 
259              print '  <p,q> = %f' % matrixmultiply(p,q) 
260              print '  |Aq| = %f' % linalg.norm(matrixmultiply(J_coords,q)) 
261              print '  |transpose(A)p| = %f\n' % linalg.norm(matrixmultiply(transpose(J_coords),p)) 
262  
 
263          self.info(C, -1) 
264  
 
265          return True

266  
 


267 -    def info(self, C, ind=None):


268          if ind is None: 
269              ind = range(len(self.found)) 
270          elif isinstance(ind, int): 
271              ind = [ind] 
272  
 
273          strlist = [] 
274          for n, i in enumerate(ind): 
275              strlist.append('a = ' + repr(self.found[i].a)) 
276  
 
277          BifPoint.info(self, C, ind, strlist)

278  
 
279  
 
280  
 


281 -class HopfPoint(BifPoint):


282 -    def __init__(self, testfuncs, flagfuncs, stop=False):


283          BifPoint.__init__(self, testfuncs, flagfuncs, 'H', stop=stop)

284  
 


285 -    def process(self, X, V, C):


286          """Tolerance for eigenvalues a possible problem when checking for neutral saddles.""" 
287          BifPoint.process(self, X, V, C) 
288  
 
289          J_coords = C.CorrFunc.jac(X, C.coords) 
290          eigs, LV, RV = linalg.eig(J_coords,left=1,right=1) 
291  
 
292          # Check for neutral saddles
 
293          found = False 
294          for i in range(len(eigs)): 
295              if abs(imag(eigs[i])) < 1e-5: 
296                  for j in range(i+1,len(eigs)): 
297                      if C.verbosity >= 2: 
298                          if abs(eigs[i]) < 1e-5 and abs(eigs[j]) < 1e-5: 
299                              print 'Fold-Fold point found in Hopf!\n' 
300                          elif abs(imag(eigs[j])) < 1e-5 and abs(real(eigs[i]) + real(eigs[j])) < 1e-5: 
301                              print 'Neutral saddle found!\n' 
302              elif abs(real(eigs[i])) < 1e-5: 
303                  for j in range(i+1, len(eigs)): 
304                      if abs(real(eigs[j])) < 1e-5 and abs(real(eigs[i]) - real(eigs[j])) < 1e-5: 
305                          found = True 
306                          w = abs(imag(eigs[i])) 
307                          if imag(eigs[i]) > 0: 
308                              p = conjugate(LV[:,j])/linalg.norm(LV[:,j]) 
309                              q = RV[:,i]/linalg.norm(RV[:,i]) 
310                          else: 
311                              p = conjugate(LV[:,i])/linalg.norm(LV[:,i]) 
312                              q = RV[:,j]/linalg.norm(RV[:,j]) 
313  
 
314          if not found: 
315              del self.found[-1] 
316              return False 
317  
 
318          direc = conjugate(1/matrixmultiply(conjugate(p),q)) 
319          p = direc*p 
320  
 
321          # Alternate way to compute 1st lyapunov coefficient (from Kuznetsov [4])
 
322  
 
323          #print (1./(w*w))*real(1j*matrixmultiply(conjugate(p),b1)*matrixmultiply(conjugate(p),b3) + \
 
324          #   w*matrixmultiply(conjugate(p),trilinearform(D,q,q,conjugate(q))))
 
325  
 
326          self.found[-1].w = w 
327          self.found[-1].l1 = firstlyapunov(X, C.CorrFunc, w, J_coords=J_coords, p=p, q=q, check=(C.verbosity==2)) 
328          self.found[-1].eigs = eigs 
329  
 
330          self.info(C, -1) 
331  
 
332          return True

333  
 


334 -    def info(self, C, ind=None):


335          if ind is None: 
336              ind = range(len(self.found)) 
337          elif isinstance(ind, int): 
338              ind = [ind] 
339  
 
340          strlist = [] 
341          for n, i in enumerate(ind): 
342              strlist.append('w = ' + repr(self.found[i].w)) 
343              strlist.append('l1 = ' + repr(self.found[i].l1)) 
344  
 
345          BifPoint.info(self, C, ind, strlist)

346  
 
347  
 
348  # Codimension-2 bifurcations
 
349  
 


350 -class BTPoint(BifPoint):


351 -    def __init__(self, testfuncs, flagfuncs, stop=False):


352          BifPoint.__init__(self, testfuncs, flagfuncs, 'BT', stop=stop)

353  
 


354 -    def process(self, X, V, C):


355          BifPoint.process(self, X, V, C) 
356  
 
357          J_coords = C.CorrFunc.sysfunc.jac(X, C.coords) 
358  
 
359          W, VL, VR = linalg.eig(J_coords, left=1, right=1) 
360  
 
361          self.found[-1].eigs = W 
362  
 
363          if C.verbosity >= 2: 
364              if C.CorrFunc.testfunc.data.B.shape[1] == 2: 
365                  b = matrixmultiply(transpose(J_coords), C.CorrFunc.testfunc.data.w[:,0]) 
366                  c = matrixmultiply(J_coords, C.CorrFunc.testfunc.data.v[:,0]) 
367              else: 
368                  b = C.CorrFunc.testfunc.data.w[:,0] 
369                  c = C.CorrFunc.testfunc.data.v[:,0] 
370              print '\nChecking...' 
371              print '  <b,c> = %f' % matrixmultiply(transpose(b), c) 
372              print '\n' 
373  
 
374          self.info(C, -1) 
375  
 
376          return True

377  
 


378 -    def info(self, C, ind=None):


379          if ind is None: 
380              ind = range(len(self.found)) 
381          elif isinstance(ind, int): 
382              ind = [ind] 
383  
 
384          BifPoint.info(self, C, ind)

385  
 
386  
 
387  
 


388 -class ZHPoint(BifPoint):


389 -    def __init__(self, testfuncs, flagfuncs, stop=False):


390          BifPoint.__init__(self, testfuncs, flagfuncs, 'ZH', stop=stop)

391  
 


392 -    def process(self, X, V, C):


393          BifPoint.process(self, X, V, C) 
394  
 
395          J_coords = C.CorrFunc.sysfunc.jac(X, C.coords) 
396  
 
397          W, VL, VR = linalg.eig(J_coords, left=1, right=1) 
398  
 
399          self.found[-1].eigs = W 
400  
 
401          self.info(C, -1) 
402  
 
403          return True

404  
 


405 -    def info(self, C, ind=None):


406          if ind is None: 
407              ind = range(len(self.found)) 
408          elif isinstance(ind, int): 
409              ind = [ind] 
410  
 
411          BifPoint.info(self, C, ind)

412  
 
413  
 
414  
 


415 -class CPPoint(BifPoint):


416 -    def __init__(self, testfuncs, flagfuncs, stop=False):


417          BifPoint.__init__(self, testfuncs, flagfuncs, 'CP', stop=stop)

418  
 


419 -    def process(self, X, V, C):


420          BifPoint.process(self, X, V, C) 
421  
 
422          J_coords = C.CorrFunc.sysfunc.jac(X, C.coords) 
423          B = C.CorrFunc.sysfunc.hess(X, C.coords, C.coords) 
424  
 
425          W, VL, VR = linalg.eig(J_coords, left=1, right=1) 
426  
 
427          q = C.CorrFunc.testfunc.data.C/linalg.norm(C.CorrFunc.testfunc.data.C) 
428          p = C.CorrFunc.testfunc.data.B/matrixmultiply(transpose(C.CorrFunc.testfunc.data.B),q) 
429  
 
430          self.found[-1].eigs = W 
431  
 
432          a = 0.5*matrixmultiply(transpose(p), reshape([bilinearform(B[i,:,:], q, q) \
 
433                  for i in range(B.shape[0])],(B.shape[0],1)))[0][0] 
434          if C.verbosity >= 2: 
435              print '\nChecking...' 
436              print '  |a| = %f' % a 
437              print '\n' 
438  
 
439          self.info(C, -1) 
440  
 
441          return True

442  
 


443 -    def info(self, C, ind=None):


444          if ind is None: 
445              ind = range(len(self.found)) 
446          elif isinstance(ind, int): 
447              ind = [ind] 
448  
 
449          BifPoint.info(self, C, ind)

450  
 
451  
 
452  
 


453 -class DHPoint(BifPoint):


454 -    def __init__(self, testfuncs, flagfuncs, stop=False):


455          BifPoint.__init__(self, testfuncs, flagfuncs, 'DH', stop=stop)

456  
 


457 -    def process(self, X, V, C):


458          BifPoint.process(self, X, V, C) 
459  
 
460          J_coords = C.CorrFunc.sysfunc.jac(X, C.coords) 
461          eigs, LV, RV = linalg.eig(J_coords,left=1,right=1) 
462  
 
463          self.found[-1].eigs = eigs 
464  
 
465          self.info(C, -1) 
466  
 
467          return True

468  
 


469 -    def info(self, C, ind=None):


470          if ind is None: 
471              ind = range(len(self.found)) 
472          elif isinstance(ind, int): 
473              ind = [ind] 
474  
 
475          BifPoint.info(self, C, ind)

476  
 
477  
 
478  
 


479 -class GHPoint(BifPoint):


480 -    def __init__(self, testfuncs, flagfuncs, stop=False):


481          BifPoint.__init__(self, testfuncs, flagfuncs, 'GH', stop=stop)

482  
 


483 -    def process(self, X, V, C):


484          BifPoint.process(self, X, V, C) 
485  
 
486          J_coords = C.CorrFunc.sysfunc.jac(X, C.coords) 
487          eigs, LV, RV = linalg.eig(J_coords,left=1,right=1) 
488  
 
489          # Check for neutral saddles
 
490          found = False 
491          for i in range(len(eigs)): 
492              if abs(imag(eigs[i])) < 1e-5: 
493                  for j in range(i+1,len(eigs)): 
494                      if C.verbosity >= 2: 
495                          if abs(eigs[i]) < 1e-5 and abs(eigs[j]) < 1e-5: 
496                                  print 'Fold-Fold point found in Hopf!\n' 
497                          elif abs(imag(eigs[j])) < 1e-5 and abs(real(eigs[i]) + real(eigs[j])) < 1e-5: 
498                                  print 'Neutral saddle found!\n' 
499              elif abs(real(eigs[i])) < 1e-5: 
500                  for j in range(i+1, len(eigs)): 
501                      if abs(real(eigs[j])) < 1e-5 and abs(real(eigs[i]) - real(eigs[j])) < 1e-5: 
502                          found = True 
503                          w = abs(imag(eigs[i])) 
504                          if imag(eigs[i]) > 0: 
505                              p = conjugate(LV[:,j]/linalg.norm(LV[:,j])) 
506                              q = RV[:,i]/linalg.norm(RV[:,i]) 
507                          else: 
508                              p = conjugate(LV[:,i]/linalg.norm(LV[:,i])) 
509                              q = RV[:,j]/linalg.norm(RV[:,j]) 
510  
 
511          if not found: 
512              del self.found[-1] 
513              return False 
514  
 
515          direc = conjugate(1/matrixmultiply(conjugate(p),q)) 
516          p = direc*p 
517  
 
518          # Alternate way to compute 1st lyapunov coefficient (from Kuznetsov [4])
 
519  
 
520          #print (1./(w*w))*real(1j*matrixmultiply(conjugate(p),b1)*matrixmultiply(conjugate(p),b3) + \
 
521          #   w*matrixmultiply(conjugate(p),trilinearform(D,q,q,conjugate(q))))
 
522  
 
523          self.found[-1].w = w 
524          self.found[-1].l1 = firstlyapunov(X, C.CorrFunc.sysfunc, w, J_coords=J_coords, p=p, q=q, check=(C.verbosity==2)) 
525          self.found[-1].eigs = eigs 
526  
 
527          self.info(C, -1) 
528  
 
529          return True

530  
 


531 -    def info(self, C, ind=None):


532          if ind is None: 
533              ind = range(len(self.found)) 
534          elif isinstance(ind, int): 
535              ind = [ind] 
536  
 
537          strlist = [] 
538          for n, i in enumerate(ind): 
539              strlist.append('w = ' + repr(self.found[i].w)) 
540              strlist.append('l1 = ' + repr(self.found[i].l1)) 
541  
 
542          BifPoint.info(self, C, ind, strlist)

543  
 
544  
 
545  
 
546  # Discrete maps
 
547  
 


548 -class LPCPoint(BifPoint):


549 -    def __init__(self, testfuncs, flagfuncs, stop=False):


550          BifPoint.__init__(self, testfuncs, flagfuncs, 'LPC', stop=stop)

551  
 


552 -    def process(self, X, V, C):


553          BifPoint.process(self, X, V, C) 
554  
 
555          J_coords = C.sysfunc.jac(X, C.coords) 
556  
 
557          W, VL, VR = linalg.eig(J_coords, left=1, right=1) 
558  
 
559          self.found[-1].eigs = W 
560  
 
561          self.info(C, -1) 
562  
 
563          return True

564  
 


565 -    def info(self, C, ind=None):


566          if ind is None: 
567              ind = range(len(self.found)) 
568          elif isinstance(ind, int): 
569              ind = [ind] 
570  
 
571          BifPoint.info(self, C, ind)

572  
 
573  
 
574  
 


575 -class PDPoint(BifPoint):


576 -    def __init__(self, testfuncs, flagfuncs, stop=False):


577          BifPoint.__init__(self, testfuncs, flagfuncs, 'PD', stop=stop)

578  
 


579 -    def process(self, X, V, C):


580          """Do I need to compute the branch, or will it always be in the direction of freepar = constant?""" 
581          BifPoint.process(self, X, V, C) 
582  
 
583          F = DiscreteMap(C.sysfunc, period=2*C.sysfunc.period) 
584          FP = FixedPointMap(F) 
585  
 
586          J_coords = FP.jac(X, C.coords) 
587          J_params = FP.jac(X, C.params) 
588  
 
589          # Locate branch of double period map
 
590          W, VL = linalg.eig(J_coords, left=1, right=0) 
591          ind = argsort([abs(eig) for eig in W])[0] 
592          psi = real(VL[:,ind]) 
593  
 
594          A = r_[c_[J_coords, J_params], [V]] 
595          W, VR = linalg.eig(A) 
596          W0 = argsort([abs(eig) for eig in W])[0] 
597          V1 = real(VR[:,W0]) 
598  
 
599          H = FP.hess(X, C.coords+C.params, C.coords+C.params) 
600          c11 = matrixmultiply(psi,[bilinearform(H[i,:,:], V, V) for i in range(H.shape[0])]) 
601          c12 = matrixmultiply(psi,[bilinearform(H[i,:,:], V, V1) for i in range(H.shape[0])]) 
602          c22 = matrixmultiply(psi,[bilinearform(H[i,:,:], V1, V1) for i in range(H.shape[0])]) 
603  
 
604          beta = 1 
605          alpha = -1*c22/(2*c12) 
606          V1 = alpha*V + beta*V1 
607          V1 /= linalg.norm(V1) 
608  
 
609          J_coords = C.sysfunc.jac(X, C.coords) 
610          W = linalg.eig(J_coords, right=0) 
611  
 
612          self.found[-1].eigs = W 
613          self.found[-1].branch_period = 2*C.sysfunc.period 
614          self.found[-1].branch = todict(C, V1) 
615  
 
616          self.info(C, -1) 
617  
 
618          return True

619  
 


620 -    def info(self, C, ind=None):


621          if ind is None: 
622              ind = range(len(self.found)) 
623          elif isinstance(ind, int): 
624              ind = [ind] 
625  
 
626          strlist = [] 
627          for n, i in enumerate(ind): 
628              strlist.append('Period doubling branch angle = ' + repr(matrixmultiply(tocoords(C, self.found[i].V), \
 
629                  tocoords(C, self.found[i].branch)))) 
630  
 
631          BifPoint.info(self, C, ind, strlist)

632  
 
633  
 
634  
 


635 -class NSPoint(BifPoint):


636 -    def __init__(self, testfuncs, flagfuncs, stop=False):


637          BifPoint.__init__(self, testfuncs, flagfuncs, 'NS', stop=stop)

638  
 


639 -    def process(self, X, V, C):


640          BifPoint.process(self, X, V, C) 
641  
 
642          J_coords = C.sysfunc.jac(X, C.coords) 
643  
 
644          eigs, VL, VR = linalg.eig(J_coords, left=1, right=1) 
645  
 
646          # Check for nonreal multipliers
 
647          found = False 
648          for i in range(len(eigs)): 
649              for j in range(i+1,len(eigs)): 
650                  if imag(eigs[i]) > 1e-5 and imag(eigs[j]) > 1e-5 and abs(eigs[i]*eigs[j] - 1) < 1e-5: 
651                      found = True 
652  
 
653          if not found: 
654              del self.found[-1] 
655              return False 
656  
 
657          self.found[-1].eigs = eigs 
658  
 
659          self.info(C, -1) 
660  
 
661          return True

662  
 


663 -    def info(self, C, ind=None):


664          if ind is None: 
665              ind = range(len(self.found)) 
666          elif isinstance(ind, int): 
667              ind = [ind] 
668  
 
669          BifPoint.info(self, C, ind)

670
```

  


| Home | Trees | Indices | Help | PyDSTool |
| --- | --- | --- | --- | --- |

|  |  |
| --- | --- |
| Generated by Epydoc 3.0.1 on Fri May 4 15:24:22 2012 | http://epydoc.sourceforge.net |
